# System within systems: challenges and opportunities for the Expanded Programme on Immunisation in Pakistan

**DOI:** 10.1186/s12961-019-0452-z

**Published:** 2019-05-17

**Authors:** Zaeem Haq, Babar Tasneem Shaikh, Nhan Tran, Assad Hafeez, Abdul Ghaffar

**Affiliations:** 1 Health Services Academy, Chak Shahzad, Park Road, Islamabad, 44000 Pakistan; 2 Alliance for Health Policy & Systems Research, Geneva, Switzerland

**Keywords:** Immunisation programme, Pakistan, challenges, health system, governance

## Abstract

**Background:**

Pakistan has one of the highest infant and child mortality rates in the world, half of these occurring due to vaccine-preventable diseases. The country started its Expanded Programme on immunisation (EPI) in 1978. However, the programme’s performance is often questioned, as the Immunisation rates have been chronically low and on-time vaccination unsatisfactory. We explored the programme’s insights about its structural and implementation arrangements within the larger governance system, and the ensuing challenges as well as opportunities.

**Methods:**

We carried out a qualitative case study comprised of semi-structured, in-depth interviews with 34 purposively selected key informants from various tiers of immunisation policy and programme implementation. The interviews revolved around WHO’s six building blocks of a health system, their interactions with EPI counterparts, and with the outer ecological factors. Interviews were transcribed and content analysed for emergent themes.

**Results:**

The EPI faces several challenges in delivering routine immunisation (RI) to children, including lack of clarity on whether to provide vaccination through fixed centres or mobile teams, scarcity of human resource at various levels, lack of accurate population data, on-ground logistic issues, lack of a separate budget line for EPI, global pressure for polio, less priority to prevention by the policy, security risks for community-based activities, and community misconceptions about vaccines.

**Conclusions:**

The fulcrum for most of the challenges lies where EPI service delivery interacts with components of the broader health system. The activities for polio eradication have had implications for RI. Socio-political issues from the national and global environment also impact this system. The interplay of these factors, while posing challenges to effective implementation of RI, also brings opportunities for improvement. Collective effort from local, national and global stakeholders is required for improving the immunisation status of Pakistani children, global health security and the sustainable development goals.

**Electronic supplementary material:**

The online version of this article (10.1186/s12961-019-0452-z) contains supplementary material, which is available to authorized users.

## Background

Preventing disease through various means, including by vaccination, is an important part of the global development agenda, articulated as the Sustainable Development Goals [1]. Under Sustainable Development Goal 3, which deals with health, a specific target has been set for providing access to affordable essential medicines and vaccines, especially in developing countries [2]. With only two-thirds of children receiving full immunisation [3], Pakistan is among those countries where immunisation rates have been chronically low [4, 5] and those of on-time vaccination even lower [6]. Additionally, a wide disparity exists between better served urban and poorly served, rural areas [5, 7]. The country is still struggling with polio and has long been the focus of attention, along with Afghanistan and Nigeria, for polio eradication [8].

Pakistan initiated its Expanded Programme on Immunisation (EPI) in 1978. Through the past decades, the programme has managed 7000 fixed centres for vaccination that contributed to improved immunisation rates, albeit slowly [9, 10]. Following the 18th constitutional amendment in 2010, health was devolved to provinces [11]; the federal EPI was assigned fewer activities, such as the purchasing of vaccines, while provinces were tasked with immunisation service delivery. The country launched the Polio Eradication Initiative in 1994 and added, from 2000 onwards, supplemental activities like national immunisation days multiple times a year [12]. In 2014, polio was declared a national emergency and a high-level Emergency Operations Centre (EOC) was constituted. The EOC works under the office of the Prime Minister, with a similar set up inside Chief Ministers’ and Deputy Commissioners’ offices at provincial and district level, respectively [13].

More has been written about polio and the challenges that its eradication faces in Pakistan, rather than on routine immunisation (RI) and the ways to improve it [14, 15]. Few studies that focused on RI are commentaries analysing secondary data on the immunisation delivery system [9, 10, 16, 17]. The system level barriers highlighted by these studies include negligible contribution from the private sector, policy issues, e.g. public–private partnership decisions, programme structure and management, governance and capacity, issues in vaccine logistics, human resource deficiencies, old information systems, and an inability to address community perceptions especially of uneducated and low-income groups [9, 16, 17].

In a country where immunisation rates have chronically stagnated, and where 50% of post-neonatal deaths are attributed to vaccine-preventable diseases [18], more frequent and deeper examination of the delivery system is required. Review of the literature, however, reveals much less propensity of scientists towards examining the system or its supply side [10, 16, 19] than on surveying demand side and testing educational interventions [7, 20–27] aimed at improving behaviours. Importantly, despite recommendations to place the health workforce at the centre of a system [28], only one study [29] explored the views of programme staff to understand the situation and improve strategies in the country.

The present study fills this knowledge gap by exploring the views of policy-makers, managers and staff working at different levels of immunisation delivery in the country. The specific objectives of this exploration include (1) understanding the building blocks of the immunisation delivery system and their relationship with those of the wider health system; (2) examining how the factors from the outer social ecology impact this service delivery; and (3) documenting the challenges as well as opportunities that arise from these complex relationships. This vital information gained from the system-insiders will help public and global health authorities in making decisions to improve immunisation delivery, prevent childhood diseases, and reduce threats to national and global health security.

## Methods

### Conceptual framework

The concept of six building blocks proposed by WHO is an insightful way of examining a health system. These building blocks include service delivery, health workforce, health information system, access to essential medicines, financing, and governance and leadership [30]. The framework is helpful in providing a common language to discussions about health systems. Some have also critiqued that it oversimplifies phenomena that are complex and have a dynamic relationship with each other and with the world outside of this system [31]. Additionally, immunisation programmes, in general, exist as vertical programmes in most of countries, having implications – both positive and negative – for the larger health system [32]. In this background, it is recommended that researchers should integrate the missing ‘demand’ component and incorporate an overarching, holistic health systems viewpoint, including interaction between various elements [31].

**Fig. 1:**
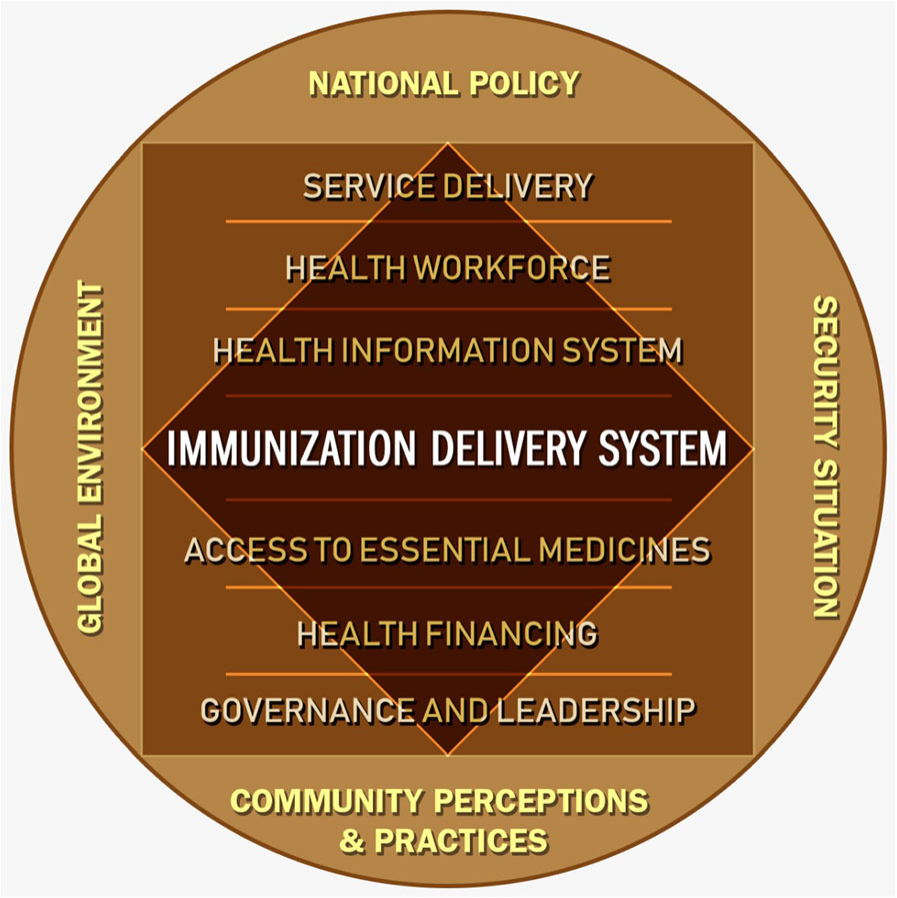
Conceptual diagram to examine relationship of six building blocks of immunisation delivery with the broader governance system

Based on these observations, we proposed that the EPI programme of Pakistan, with its own set of six building blocks, operates within the building blocks of the larger health system, both having a continuous interaction with each other. Moreover, these two also have a dynamic interface with the outer ecology, comprised of global, national, local and community factors (Fig. 1). Global pressure to eradicate polio [33], national policy that prioritised polio and acknowledged RI much later [34], Pakistan’s on-going war on terror because of which an additional layer of security became essential for immunisation workers [15, 35], and community perceptions and vaccine hesitancy [23, 36] are examples of these ecological factors. Exploring the views of immunisation programme staff in the context of these building blocks interacting with each other, and with the larger ecology, can help us understand the barriers as well as facilitators to the programme implementation. Our intention was not to apply or validate the concept of building blocks; hence, we used the concept only for developing the interview questions for this study.

### Study design, participants and setting

We carried out a qualitative case study to document the views of immunisation programme stakeholders working at different levels of policy and programme implementation in the country. Case study research is an approach through which the investigator explores a real-life, contemporary bounded system over time, through detailed, in-depth data collection involving multiple sources [37]. Based on our past experience of such studies, individual interviews were deemed appropriate, as our participants were busy professionals; one-on-one meetings in their office being the most feasible setting [38]. The research team purposively selected the participants. The inclusion criteria were participant involvement with and experience of policy development, programme management, and implementation at various levels. We invited all those individuals from the National Health Ministry, provincial Departments of Health, federal, provincial and regional EPI programmes, the development sector, and civil society, including WHO, UNICEF, USAID, John Snow Inc., Jhpeigo, and Rural Support Programme Network, who befitted our criteria of selection. Due to time and resource constraints, the district-level operators and frontline workers were not included. We invited a total of 38 and interviewed 34 key informants (Table 1) who agreed to participate. The reasons for refusal included being extremely busy in the office or out of the country.

**Table 1:** Study participants and their characteristics

Category	Number (%)	Participant characteristics
Federal	6 (18%)	Director General Health, National manager EPI and EPI team members
Provincial/Regional	14 (41%)	Team members at provincial directorate health, managers and team members of EPI and Polio programmes
Academia/Research	6 (18%)	Researchers and administrators from public and private research and academic organisations
Civil society, Development partners	8 (23%)	Members from donor and advocacy organisations, technical assistance teams

### Data collection, processing and analysis

We developed a semi-structured interview guide based on the conceptual framework (Fig. 1) for this study. The questions in this guide explored the views of participants about the six building blocks of the EPI programme, the interface of EPI building blocks with those of the broader health system, and the interaction with outer ecological factors that may have an impact on implementation of the EPI programme (Additional file 1). The interview guide was refined based on an initial four interviews. Using this guide, two members (ZH and BS) of the study team carried out interviews, one asking questions while the other took detailed notes. The interviews were conducted by visiting the office of the participant or through a phone call. Verbal consent was obtained, research objectives explained, and confidentiality discussed before the interview. The note-taker took detailed, verbatim notes of the discussions. The interviews were conducted using a mix of Urdu and English languages, with a duration ranging from 30 to 90 min. Since we were interviewing a small sample of officials working at various hierarchical levels, we decided to interview all of them and not wait for data saturation, which is the usual practice in qualitative studies [39].

Detailed notes from the interviews were the data used for analysis. An initial code list, derived from the concepts displayed in our conceptual framework, was developed for analysis. Using this code list, two members of the study team (ZH and BS) independently manually coded four initial transcripts. Following this, they met to discuss the concordance between their coding. Points where their codes did not match were debated and a consensus reached. This helped in the development of a final code sheet that was used for coding and identifying significant statements across all transcripts [39]. These meaning statements were clubbed together to identify subthemes, which were ultimately grouped under the ten themes. While we gave weightage to recurring themes, we also paid attention to the divergent themes, or points that were not shared by the majority but appeared significant [40]. Where required, the interviewee was contacted verbally to clarify whether our understanding of their opinion was correct or not. The synthesised findings were also presented to the participants in a workshop setting held in Islamabad during February 2017 to ensure member checking [41].

The study team was comprised of researchers having formal training in qualitative methodology and programme research, with significant experience of exploring the implementation issues of preventive programmes. To ensure uninhibited expression of opinions, only those members of the research team (ZH and BS) who did not have any interface with the EPI programme or health department at any level conducted the interviews. Following the interviews, all team members met to discuss the proceedings, examined field memos, and brainstormed the future course of action (e.g. any change to the guiding questions) of the study. No incentives or payments were offered to the respondents. Ethical clearance was obtained from the Institutional Review Board of the Health Services Academy Islamabad, Pakistan.

## Results

A total of 34 key informants participated in this study (Table 1), including officials from the Ministry of National Health Services, Regulation and Coordination and provincial Departments of Health, federal and provincial EPI managers from all five provinces and regions of the country and key informants belonging to partner organisations. Main themes emerging from these discussions are summarised below. In support of our structural interpretations of what participants said, we use textural descriptions (quotes in italic text) to substantiate the point [42].

### Service delivery

The participants discussed several challenges currently being faced by the immunisation service delivery. Foremost is the confusion of whether to provide vaccination at fixed centres or through mobile outreach activities. According to a participant from Punjab, “*The immunisation services provided through outreach are costly and cannot last for long, but the difficulty is that after many years of door-to-door campaigning for polio, people expect that all immunisation will be delivered at their doorstep.*” Agreeing to this, others also expressed that both options need a forthcoming involvement of the health staff outside of EPI. According to a participant from Khyber Pakhtunkhwa (KP) province, “*Health centres that serve as fixed sites have only one or two positions of vaccinator versus a bigger number of other staff (e.g. doctors, paramedics), whose conduct towards patient impacts community’s perception of quality of services, including immunisation*. *For outreach, likewise, the vaccinator needs support from LHW* [Lady Health Worker] *to mobilise families for getting their child vaccinated at the outreach point.*”

Inequity in service delivery was also mentioned as a major challenge. Despite efforts, EPI is unable to plan for and provide services to the mobile, marginalised and hard-to-reach populations. Referring to districts from southern Punjab and KP, a participant from federal EPI said, “*Immunisation service delivery to the marginalised, to populations in hard to reach areas, people in urban and peri-urban slums and nomadic population has been a critical issue due to lack of proper planning.*” A closely connected point, which is also a solution, is the issue of population estimates. The EPI programme requires accurate information of the target population at the district (and below) level to rationalise the number of outreach teams. Providing this information falls outside the scope of EPI, and mainly with the census organisation. “*Our estimates of populations, required amount of vaccines and personnel that will provide vaccination services are based on estimated additions to the number of population that we have from 1998 census. This estimation may be highly flawed leading to inadequacies in service delivery*”, shared a participant from KP.

### Health workforce

There are approximately 7000 fixed centres to provide EPI services to the target populations. Participants from provincial level thought there is a significant mal-distribution of vaccinators at the facility and community level, and additional vaccinators, especially females, are required. Their main concern, however, was about the lack of capacity-building mechanisms for the foot soldiers of immunisation. “*Although EPI policy mentions the number of vaccinator per union, there is no clarity about the on-going training, capacity-building and results-based monitoring. Only one-time training of vaccinators provided at the time of induction is not enough; refresher trainings are required. The lack of performance reviews of the vaccinators and other staff is a key bottleneck and needs to be examined*”, expressed a participant from Sindh province*.*

Lack of human resources and capacity appears even bigger at the federal and provincial levels. Managers at these levels have gigantic tasks, including country- or province-wide planning, procurement, logistics, leveraging funds, addressing community perceptions and managing hostile media. Inadequate number of staff and capacities at these levels pose huge impediments. Coupled with this is a dearth of succession planning and recurrent transfers within management tiers, that lead to a loss of institutional memory and limited levels of knowledge transfer. A participant from the federal level suggested, “*A provincial situational analysis led by health department along with provincial programmes is necessary to document the required capacities at respective levels including a HR* [Human Resources] *registry mechanism at district and provincial level to inform a robust and dynamic HR strategy.*”

### Health information system

Almost all participants talked about issues in data along with room for improvement in the data credibility and accountability. The current lack of accurate information about the target number of children leads to inaccurate planning, false reporting and pilferage of resources. A participant from KP said, “*In the absence of census during past 20 years, no accurate number is available about the children from a catchment area. As a result the planners are always uncertain about the denominator because of which the percentage of children covered by immunisation services are never reliable.*” They suggested a high level unit along the lines of a Prime Minister’s polio monitoring cell, with adequate technical depth to monitor and analyse the data at union council, district, provincial and national level.

Even if fresh census data are available, there is still a need for a mechanism to carry out ongoing micro-censuses to accurately update the number of newborn children. This is being done by community-based vaccinators in 11 high-priority districts for polio, and should be achieved for RI through Lady Health Workers, who work throughout the country. “*There is no single and comprehensive source of family data that vaccinator can use to plan their demand and ultimate delivery of the vaccine. Micro-census in both urban and rural areas is the solution to ascertain the correct denominator of children to be vaccinated*”, was the statement of a participant from KP.

### Essential medicines (vaccines and supplies)

While most of the participants from higher levels opined that vaccine supply and availability is satisfactory, those dealing at the district (and below) level did not completely agree to it. According to them, stock-outs do occur due to inaccurate estimations and cumbersome procedures. For example, a participant from Balochistan province shared, “*Looking from the top, supply of vaccines may seem fine but stock-outs ranging from one to three months do occur at district and below level, mainly due to budget deficit for vehicle maintenance and fuel.*”

They also highlighted the importance of accurate recording and reporting of vaccine availability, its storage, utilisation and wastage, citing a few examples where vaccines were apparently being consumed but not actually administered. “*In the absence of accurate population estimates, the data about forecasting of vaccines and their use becomes flawed. At places, the targets are achieved but there still are a significant number of children waiting to be vaccinated. At others, vaccines get expired and dumped inappropriately as enough number of children are not found*”, was shared by a participant from federal level.

Another point was about the devolved responsibilities, time when vaccine procurement will become a provincial responsibility. If procurement is to devolve as suggested, an important question is whether provinces are prepared regarding procedures and whether the federal government has informed them about these, along with providing technical assistance and necessary infrastructure. “*It is important for the higher levels to assess whether the provincial and district storage capacity is up to the mark, whether back-up plans for power failures are available and working at all health facilities, and when and how the logistics and vaccine management software can be introduced at provincial and district levels*”, was the concern shared by a participant from Sindh province.

### Health financing

The Government of Pakistan, with assistance from development partners, finances the immunisation services in the country. The bulk of resources come from GAVI/UNICEF for purchasing vaccines, while the government takes care of salaries of EPI staff, supplies and stationery, and other requirements like vehicle fuel and maintenance. Bottlenecks are usually faced in securing the government’s share. A focal person for district monitoring from Balochistan province shared, “*Under the current budgetary arrangements, shortages in fuel and repair or maintenance budgets frequently occur, leading to restricted field visits of vaccinators and the monitoring staff. Likewise, immunisation cards are usually short in supply and as a replacement, the date for next visit is written on a small piece of paper and handed over to parents.*”

The budgetary issues and their solutions lie with the broader governance system. The annual budgetary allocations for health are approved by legislature, handed over to provincial health department, and ultimately to the District Health Officer (DHO). The DHO has to release funds for EPI from the same kitty, where different priorities compete with each other, leading to slowing down of the process. A separate budget line for EPI can help in rapid transfer of funds through all levels. “*Look at the innumerable responsibilities of a DHO and the priority that curative services usually take over the preventive programmes. The solution is a separate budget line for preventive programmes including EPI. This decision of creating a separate budget line, however, depends on provincial minister and secretary, EPI having no say in this decision*”, was the submission of a participant from the Punjab province.

### Governance and leadership

The participants discussed the overall structure of the immunisation programme organised within the wider governance system. They also discussed the position and power of different actors, placed at different hierarchical levels. “*Bottlenecks in governance include the lack of transparency, accountability and regular programme reviews. When it comes to accountability, the performance of vaccinator is usually mentioned, while that of higher levels, tasked with programme planning and capacity-building, is conveniently ignored. At the same time, it is also true that federal and provincial levels have a lack of human resource and capacity and the increase in number of key positions at these levels is the mandate of wider system and not the EPI*”, was a sentiment shared by a participant from KP.

Coordination issues seemed particularly important to participants when they explained governance challenges that emerge from the implementation of RI and polio eradication through two separate arms of the same programme. Polio eradication, that works under EOC and not EPI at all administrative levels, uses the EPI workforce but has its own administrative, financing and reporting structure. “*It is said that polio programme has developed better monitoring and accountability mechanisms. This may be true but the fact remains that being an offshoot, it’s mechanisms cannot perform the accountability of the parent programme, on their own. There has to be a policy direction from the health ministry or department – the creator and regulator of both these programmes*”, suggested a member of a donor agency.

The participants were unanimous in that governance issues cannot be addresses by the same level of operations that created them and only a mechanism from higher levels can help address these issues. It is important to define the role of the national ministry and strengthen the mechanisms for inter-provincial coordination to improve the overall performance and integration of the immunisation services in the country. “*Federal EPI cell and interprovincial coordination committee with representation from all provinces and regions should work together. A donor coordination forum for immunisation needs to be established with defined scope and responsibilities. Higher policy should provide directions for synergies between polio and EPI, and collaborating with private sector to cover huge discrepancies in vaccine coverage*”, were the key steps suggested by a participant from Balochistan.

### Global environment

The participants shared how some of the global factors impact the immunisation system in Pakistan. Two of these, i.e. global interest in polio eradication and the war on terror, were most commonly mentioned. The global players have more interest in Pakistan’s polio eradication and, as a result, the political push and financial assistance for polio has been much more than for RI in Pakistan. This became highly intensified when polio showed a surge in 2014 and sanctions according to international health regulations were imposed on Pakistan. A participant from KP said, “*In 2014, the growth in polio cases in Pakistan generated much discussion and debate ultimately causing the enforcement of travel bans as per international health regulations. Identifying the severity of the situation, an emergency centre* [EOC] *was established. Polio started receiving much more funding, visibility and authority, ignoring sometimes the fact that it was a strong RI that would ultimately guarantee the sustainable eradication of polio.*”

While the participants were aware of global factors that impinge upon RI, they were equally mindful that such factors can be addressed by the wider governance because only that layer comes into contact with the global factors of security and regulatory nature. An EPI official from Punjab said, “*Health ministry and the provincial health departments, along with the wider governance system have the opportunity to sit on the negotiation table with representatives of other governments and agencies. So, they are in the best position to forewarn about consequences of bilateral decisions. Similarly, when contentious decisions like participating in a war are being made, those at the table are the first to consider the consequences that a decision will bring to their people.*”

### National policy

The participants discussed several policy issues that implicate the immunisation system. Foremost is that health policy has always had focus on ‘sickness care’ rather than ‘preventing disease’ and ‘promoting health’ in the country. Though so-called policy documents seem to show the intent of prevention and promotion, the implementation usually betrays these. “*If policy is a statement of intent, resource allocation is a practical reflection of that policy. Before devolution of health, most of public health exchequer used to be on hospitals, equipment, medicines and salaries. Almost all the preventive programmes functioned with support from international donors. This is true even today; EPI is an example where about two-thirds of programme support comes from international donors*”, shared participants both from federal and provincial level.

Our discussions with participants from various levels also provided a big picture of the post-devolution phase. There seems to be some ambiguity of the mandate that federal and provincial levels have for EPI. The roles and responsibilities of federal and provincial programmes need to be clarified, with financial layout for the next 5-year period reassessed, in the context of devolution and provincial responsibilities. “*The comprehensive Multi Year Plans along with operational plans for each year should be developed in each province to ensure better planning and implementation of the programme. There is a lack of information about the processes of allocating funds at the district level by relevant authorities, which needs to be made transparent*”, was a sentiment shared by a manager from Sindh province.

The question of how to enhance coverage through the involvement of the private sector, that currently contributes only 3% of the total immunisation, was also discussed. The challenge is that the private sector is mostly unregulated and consists of all forms of medics and healers, many of whom do not have proper training or even a practicing license. Even the formally trained and licensed practitioners are not directly engaged by EPI, as it is not mandated. “*The door-to-door micro census has often revealed families where all children had received polio drops during each campaign but had missed doses of routine immunisation because they routinely consulted a private physician who never advised about vaccinations. EPI should engage with these private physicians but being a public sector programme, it needs direction and facilitation from national ministry or provincial health departments*”, shared a provincial manager.

### Security situation

The participants explained how, in the wake of 9/11, the global war on terror and the geopolitical situation of Pakistan and Afghanistan impacted polio eradication and other immunisation efforts in the country. The country, where polio cases were surging in 2013–2014, saw a significant drop when the security situation improved along the border between Pakistan and Afghanistan. A participant from the federal ministry shared, “*Up until 2014, we were struggling with polio being reported not only from border areas but also from Karachi and other districts. Our immunisation activities were seriously affected as health workers were targeted and killed. It was in 2015 that territorial gains made by the Pakistani military in Wazirastan agency gave more access to vaccinator teams in tribal areas. The collaborations between Pakistan’s security forces and the polio teams helped achieve a reduction of over 80% in new polio cases reported that year.*”

### Community perceptions and practices

In their discussions, almost all interviewees talked about the demand-side issues and highlighted the need for addressing community misperceptions and facilitating their immunisation behaviours. According to them, it is mainly the conservative minded, blind followers of faith leaders, or nomads who usually refuse RI. “*A common feature is that these people do not usually have enough knowledge of the purpose and benefits of vaccines; providing this information usually proves helpful. The challenge is that misperceptions may not be the same across provinces and districts; hence a blanket communication is less likely to work. Exploring the local factors and perceptions that may be acting as a barrier and integrating this with other public health communication is required*”, stated a participant from federal EPI.

Participants also emphasised the importance of mass media and the need of being able to connect the locally implemented campaigns with the on-going health messages on mass media. According to them, the local and mass media components of the current communication campaigns do not complement each other, because of which the quantum of effect is lost. A participant from KP said, “*Local campaigns should address the local misperceptions while also establishing a connection with the mass media messages, which are there to sensitise the masses on importance of immunisation while reinforcing the content being disseminated at the local level, at the same time.*”

## Discussion

This study is the first in several respects. Following the three objectives that it set for itself, and using the WHO’s concept of six building blocks of a health system, it describes the complex relationship between building blocks of immunisation delivery and those of the broader health system. In addition, it sheds light on some factors from the outer, socio-political ecology that impact this system and should be considered while assessing health service delivery. It also indicates how activities for polio eradication may have implicated immunisation programming. The complex interplay of these factors poses many challenges to effective delivery of RI; however, this interplay also brings opportunities for addressing the impediments and improving immunisation delivery in the country.

Our study reiterates the earlier reported problems in the immunisation system, including gaps in service delivery [19, 43], lack of a comprehensive human resource strategy, unwelcoming attitude of staff towards clients [16] and political interference [10]. The participants of our study also reported inadequacies in data, cold chain maintenance, governance and public advocacy; all of which are problems that have been reported earlier [9, 10, 44]. In addition, our study highlights some deeper issues within these gaps. These include indecision about the service delivery points, capacity issues at higher levels of EPI, inability to gather, process and utilise population data, inept resource allocation and funds transfer, and a lukewarm approach towards governance issues and community misperceptions. Since these gaps arise at points where EPI has an interaction with the broader health and governance, their improvement too requires concerted efforts of the entire system.

For example, the debate about point of service delivery has emerged from years of door-to-door campaigns for polio, which was decided by the broader governance and has raised public expectations. A coherent policy response is now required on whether to provide resources for costly, outreach activities, or stick to fixed centres. The inadequate human resources at federal and provincial EPI is another gap that hampers planning, execution and programme development at all levels. The power of decision-making about the numbers and capacities at these levels is with the federal ministry and provincial health departments. Non-availability of resources for buying fuel and maintaining vehicles is another gap that can be simply addressed by the Finance Ministry through creating a separate budget line for EPI.

The denominator issue surfaced at several nodes of our discussions, including service delivery, information system and supply chain of vaccines. The question of likely fallacies in the ‘administrative coverage estimates’ has been raised [45] in the published literature, as have been the usual reasons including incorrect assumptions about fertility and/or mortality, and estimates of migrant populations [46]. While these challenges may hold true in Pakistan, our discussions revealed a much bigger problem of non-availability of census data during the past decade, which requires on-time execution of census – a mandate of broader governance system and not the EPI.

The interaction of immunisation programmes with the broader health system has been examined more in terms of impact of routine or supplemental (polio and measles campaigns or introduction of new vaccines) onto the mainstream healthcare delivery [47–51]. Some have extended this discussion by looking at the impact of donor-funded programmes (e.g. Global Fund) on the mainstream health system [52–54]; concerns are mostly that such programmes may (1) bring conflicting reporting lines, (2) lead managers to specifically highlight their programme’s results, (3) cause lower cadres to focus on areas where funds are located, and (4) well-funded programmes may shift attention away from ‘patient-centred’ healthcare [47]. Our study reverses the lens, indicating that certain decisions, such as providing policy on dealing with an unregulated private sector, issues emerging from devolution, negotiating local and global security, and setting local and national priorities in the wake of a global push for just one disease (e.g. polio), are the responsibility of the broader governance system.

Unger et al. [47] have described a useful typology according to which EPI seems an ‘integrated’ programme (its administrative and operational sides are integrated with the main healthcare delivery) while polio appears as an ‘indirect’ programme (administrative arm exists outside of main health system but operational side works through this system). According to their analysis, integration of preventive programmes with healthcare delivery is the most helpful strategy. Effectiveness of this strategy depends on two conditions, namely (1) the administrative and operational sides are not separate; and (2) the system does not confine disease control to the public sector and healthcare delivery to the private sector. Building on their analysis, we think that immunisation in Pakistan could be improved if EPI were to be fully integrated within the broader health system.

Our study set for itself the task of examining complex, within (building blocks of immunisation programme and broader health delivery) and between (health with the broader, national and global governance) system relationships to suggest recommendations for programme and policy. Its strengths lie in the exploration of the programme’s own perspective on the challenges it faces in the implementation as well as the collection of information from several tiers. Our conceptual framework was helpful in exploring the complex interrelationships and inferring where the implementation is facing impediments. However, we were constrained by time and resources and could not dwell more on the operational and community level exploration. The same constraint also prevented the collection of information from administrators (e.g. secretary of health and the team) of all the provincial health departments. Their views, representing the broader health system, could have provided a more comprehensive picture. Lastly, the national census was also underway, albeit with a delay of 10 years, when we conducted this study [55]. The final results from this census will be available soon and the issue of inaccuracy of population data will hopefully be addressed.

## Conclusions

Juxtaposing the six building blocks of immunisation delivery and those of the broader health system reveals an interesting relationship. The complex interplay poses many challenges to effective delivery of RI, including lack of clarity on whether to provide vaccination through fixed centres or mobile teams, scarcity of human resource at various levels, lack of accurate population data, on-ground logistic issues, lack of a separate budget line for EPI, global pressure for a focus on polio, less policy priority for prevention, security risks for community-based activities, and community misconceptions about vaccines.

The fulcrum of these challenges lies where EPI service delivery has to interact with components of the broader health system. The activities for polio eradication have also had implications for RI. Socio-political factors from the national and global environment also impact this system. The federal health ministry and provincial health departments need to realise that EPI can perform better if it is integrated into rather than vertical to the health system. The governments should take concrete steps towards ensuring integration, i.e. the administrative and operational sides of all immunisation activities should work together, as part of the broader health system. Global actors like GAVI and donor agencies should also facilitate this integration. The synergised engagement of local, national and global stakeholders can go a long way in improving the country’s immunisation system, global health security, and achievement of the sustainable development goals.

## Additional file

Additional file 1: COREQ (COnsolidated criteria for REporting Qualitative research) Checklist (PDF 310 kb)

## Abbreviations

DHO: District Health Officer
EOC: Emergency Operations Centre
EPI: Expanded Programme on Immunisation
KP: Khyber Pakhtunkhwa
RI: routine immunisation
